# Physiological roles of taurine in heart and muscle

**DOI:** 10.1186/1423-0127-17-S1-S2

**Published:** 2010-08-24

**Authors:** Stephen W Schaffer, Chian Ju Jong, Ramila KC, Junichi Azuma

**Affiliations:** 1 Department of Pharmacology, University of South Alabama, College of Medicine, Mobile, Alabama 36688, USA; 2 Clinical Evaluation of Medicines and Therapeutics, Graduate School of Pharmaceutical Sciences, Osaka University, Osaka 565-0871, Japan

## Abstract

Taurine (aminoethane sulfonic acid) is an ubiquitous compound, found in very high concentrations in heart and muscle. Although taurine is classified as an amino acid, it does not participate in peptide bond formation. Nonetheless, the amino group of taurine is involved in a number of important conjugation reactions as well as in the scavenging of hypochlorous acid. Because taurine is a fairly inert compound, it is an ideal modulator of basic processes, such as osmotic pressure, cation homeostasis, enzyme activity, receptor regulation, cell development and cell signalling. The present review discusses several physiological functions of taurine. First, the observation that taurine depletion leads to the development of a cardiomyopathy indicates a role for taurine in the maintenance of normal contractile function. Evidence is provided that this function of taurine is mediated by changes in the activity of key Ca^2+^ transporters and the modulation Ca^2+^ sensitivity of the myofibrils. Second, in some species, taurine is an established osmoregulator, however, in mammalian heart the osmoregulatory function of taurine has recently been questioned. Third, taurine functions as an indirect regulator of oxidative stress. Although this action of taurine has been widely discussed, its mechanism of action is unclear. A potential mechanism for the antioxidant activity of taurine is discussed. Fourth, taurine stabilizes membranes through direct interactions with phospholipids. However, its inhibition of the enzyme, phospholipid N-methyltransferase, alters the phosphatidylcholine and phosphatidylethanolamine content of membranes, which in turn affects the function of key proteins within the membrane. Finally, taurine serves as a modulator of protein kinases and phosphatases within the cardiomyocyte. The mechanism of this action has not been studied. Taurine is a chemically simple compound, but it has profound effects on cells. This has led to the suggestion that taurine is an essential or semi-essential nutrient for many mammals.

## Introduction

Taurine is an ubiquitous sulfur-containing, β-amino acid, which is considered an essential nutrient in some species [1]. Although it is found in high concentration in most mammalian tissues, its concentration is particularly high in cardiac and skeletal muscle. Lubec *et al.*[2] have shown that taurine slowly accumulates in the heart following intravenous administration but once taken up by the heart it turns over very slowly. Myocardial taurine content is species dependent, with levels varying from ~1.8 µg/g wet wt in the frog to ~39.4 µg/g wet wt in the mouse [3]. According to Kocsis *et al.*[3], a correlation exists between taurine levels and heart rate, with the highest taurine levels found in species with the highest heart rates. Moreover, a transmural gradient of taurine exists in the left ventricle, with the highest taurine concentrations found in the endocardium, which experiences the greatest work load. Based on this evidence alone Kocsis *et al.*[3] suggested that taurine might be linked through some mechanism to the workload of the heart.

A considerable body of evidence has been gathered on the pharmacological actions of taurine. These studies have largely focused on the cytoprotective activity of taurine. However, they provide little information on the physiological actions of taurine. Until recently, the physiological actions of taurine were studied using a model of taurine depletion mediated by taurine transport inhibitors or a model of nutritional depletion in cats. Although these studies have uncovered new actions of taurine, in the case of the transport inhibitors it is unclear if the reported actions are related to taurine depletion *per se* or the side effects of the transport inhibitors. The recent development of taurine transporter knockout models has facilitated the study of taurine deficiency in rodents and has dramatically improved the chances of definitively establishing the key physiological functions of taurine. The present review discusses the physiological roles of taurine in heart and skeletal muscle, focusing on the maintenance of contractile function, osmoregulation, conjugation, antioxidant activity, membrane stabilization and protein phosphorylation.

### Chemical reactions involving taurine

Taurine was once considered an end product of sulfur amino acid metabolism, a presumption based on the +4 oxidation state of the sulfur (present as a sulfonic acid moiety). Although it has been suggested that taurine can serve as an antioxidant, the sulfonic acid is incapable of scavenging the common oxidants, namely, superoxide, hydrogen peroxide and hydroxyl radical. Nonetheless, the amino group of taurine can neutralize hypochlorous acid, one of the reactive species generated by neutrophils. In that reaction, taurine is converted to taurine chloramine, which is less toxic than hypochlorous acid and serves as a modulator of the immune system [4]. The amino group of taurine is commonly involved in conjugation reactions. One potentially important reaction is the interaction of taurine with uridine to form 5-taurinomethyluridine, a modified base located in the wobble position of some mitochondrial t-RNAs [5,6]. This reaction has attracted considerable attention because of its potential involvement in the regulation of mitochondrial protein synthesis [7,8]. Other taurine-associated conjugation reactions play central roles in the elimination of toxins from the body [9], the metabolism of lipids (via bile acid conjugation) [10] and the activity of glycolipids [11]. However, the relative chemical inactivity of taurine makes the β-amino acid attractive as a modulator of basic processes, such as osmotic pressure, cation homeostasis, enzyme activity, receptor regulation, cell development and cell signaling.

### Is taurine required for normal contractile function of the heart and muscle?

Several species, among them cats, dogs and foxes, develop cardiomyopathies when maintained on a taurine deficient diet [12-14]. The original report attributing the development of a cardiomyopathy in cats to taurine deficiency was published in 1987 by Pion *et al.*[12]. Unlike most dilated cardiomyopathies, Pion *et al.*[12] found that the taurine deficient cardiomyopathy was reversible, responding favorably to taurine supplementation. Echocardiographic analysis revealed that the taurine deficient cardiomyopathy was characterized by reduced fractional shortening combined with an increase in left ventricular chamber dimensions and impaired response to dobutamine [15,16]. Novotny *et al.*[17] also found decreases in the rate of pressure rise (+dP/dt, a measure of contractility), reductions in the rate of pressure decline (-dP/dt, a measure of relaxation) and increases in ventricular chamber compliance in the taurine deficient heart of cats, suggesting that the taurine deficient heart is characterized by defects in both systolic and diastolic function. Also supporting the existence of a taurine deficient cardiomyopathy was the finding that rodents, which can readily synthesize taurine in the liver, develop a taurine deficient cardiomyopathy when the taurine transporter is genetically abolished [18].

Although the mechanism underlying the development of the taurine deficient cardiomyopathy has not been established, it is generally accepted that heart failure is characterized by impaired contraction arising from one or more of the following conditions: (a) diminished handling of calcium by the heart, (b) impaired calcium sensitivity of the contractile proteins, (c) loss of cardiomyocytes and (d) insufficient ATP to drive contraction. Several of these conditions are taurine dependent.

The regulation of calcium homeostasis by taurine has been extensively studied [19,20] although many of the studies examined the effect of extracellular taurine on both contractile function and [Ca^2+^]_i_[21-24]. Using a skinned fiber preparation containing permeabilized cell membrane, Steele *et al.*[25] and Galler *et al.*[26] provided the first evidence that physiological concentrations of taurine can increase Ca^2+^ sensitivity of contractile proteins and alter tension development. These effects of taurine were attributed to the modulation of sarcoplasmic reticular Ca^2+^ release, with the effect greater in preparations containing Ca^2+^ deficient sarcoplasmic reticulum, but less when the sarcoplasmic reticulum contain a high Ca^2+^ load. In contrast, acute taurine elevations exert no apparent effect on Ca^2+^ uptake by enriched sarcoplasmic reticular preparations [27-29]. However, the acute taurine studies do not rule out a role of the sarcoplasmic reticular Ca^2+^ pump in the chronic actions of taurine. Indeed, *in vivo* chronic taurine influences the activity of the sarcoplasmic reticular Ca^2+^ ATPase through at least two factors that are altered by taurine. First, recent evidence reveals that the phosphorylation of the sarcoplasmic reticular phosphoprotein, phospholamban is reduced in the taurine deficient heart [30]. Because the phosphorylation of phospholamban enhances the rate of Ca^2+^ uptake by the sarcoplasmic reticulum, it increases the rate of myocardial relaxation [31]. Second, there is an extensive literature touting the “antioxidant” activity of taurine (see below). According to Park *et al.*[32] the activity of the sarcoplasmic reticular Ca^2+^ ATPase is inhibited by oxidative stress. It is known that sarcoplasmic reticular Ca^2+^ uptake and Ca^2+^-induced Ca^2+^ release play key roles in regulating [Ca^2+^]_i_ and modulating Ca^2+^ delivery and removal from the muscle proteins. Moreover, the binding of Ca^2+^ to troponin determines the response of the muscle proteins to elevated [Ca^2+^]_i_. Therefore, it is not surprising that taurine is required for normal systolic and in diastolic function.

Several other factors might also contribute to systolic dysfunction in the taurine deficient heart. It has recently been shown in taurine transporter knockout mice that the phosphorylation of troponin I in the heart is significantly elevated [30]. Because the phosphorylation of troponin I diminishes tension development by interfering with the binding of Ca^2+^ to troponin C, the taurine deficient heart should generate less ventricular pressure [33]. This conclusion is consistent with the report by Eley* et al.*[34] showing that taurine deficiency is associated with a decrease in the sensitivity of cardiac muscle to Ca^2+^. The work of Eley *et al.*[34] and Lake [35] also show that there is a loss of myofibrils in drug-induced taurine deficient hearts, a finding consistent with the notion that taurine deficiency is linked to the loss of cardiomyocytes via apoptosis [36-38].

According to Novotny *et al.*[15-17] taurine-depleted cats exhibit both systolic dysfunction and a significant increase in left ventricular diastolic chamber compliance. This pattern is characteristic of an eccentric form of hypertrophy, in which the myocytes become thicker and longer than normal. However, in severe cases of eccentric hypertrophy the ventricular chamber dilates and the walls become thinner. Accordingly, the dilated cardiomyopathy phase of eccentric hypertrophy is usually associated with defects in systolic function while the ventricular hypertrophic cardiomyopathy phase is associated with diastolic dysfunction. Therefore, it is reasonable to expect that diastolic defects predominate during the initial stages of the taurine deficient cardiomypathy, while systolic dysfunction predominates in severe cases of the disease. Consistent with this interpretation, Schaffer *et al.*[36] found that rats treated with the taurine transporter blocker, β-alanine, only develop a mild diastolic defect characterized by impaired removal of Ca^2+^ from the cytosol and prolongation of the contractile cycle. In contrast, systolic dysfunction is the dominant defect in life-threatened taurine deficient cats [12,15,16].

Taurine also appears to be essential for normal contraction of skeletal muscle. Warskulat *et al.*[39] found that skeletal muscle function is severely impaired in taurine transporter knockout mice. Interestingly, physical exercise decreases taurine levels of skeletal muscle, an effect partially prevented by taurine administration [40,41]. Administration of taurine also improves physical endurance. In accordance with the actions of taurine in the heart, Bakker and Berg [42] reported that the effects of taurine are at least partially explained by an augmentation in sarcoplasmic reticular Ca^2+^ accumulation and release.

### Osmoregulatory activity of taurine in heart and muscle

Cells do not tolerate extreme alterations in cell size, therefore, they possess volume regulatory mechanisms to counteract the consequences of osmotic stress and normalize cell volume. Changes in taurine uptake and release contribute to the normalization of cell volume in most cell types. In the case of heart and muscle the turnover of taurine is normally very slow. Yet myocyte taurine content can be dramatically altered by changes in osmotic stress.

The identification of taurine as an osmoregulator in the heart originated with the work of Vislie and Fugelli [43], who studied the effect of osmotic stress in flounder undergoing a transition from salt to fresh water. Interestingly, they showed that the adaptation of flounder to fresh water was associated with decreases in plasma osmolality, and a corresponding decrease in myocardial taurine content. Although the handling of taurine by the heart was not evaluated by Vislie and Fugelli [43], Rasmusson *et al.*[44] reported that isolated chick cardiomyocytes exposed to hypoosmotic stress undergo cell swelling, followed by a volume regulatory event that extrudes osmolytes, such as taurine, from the cell.

In contrast to the response to hypoosmotic stress, the regulatory response to hyperosmotic stress leads to increased taurine influx into the cell [45]. In an attempt to explain this phenomenon, Ito *et al.*[46] examined the promoter region of the taurine transporter for osmotic sensitive elements. They found that the promoter region contains a transcriptional tonicity-responsive element (TonE), which along with the TonE binding protein is involved in the upregulation of the taurine transporter in the hyperosmotically stressed cardiomyocyte.

The osmoregulatory activity of taurine appears to be an important determinant of cell survival. Without specifying the mechanism underlying the cytoprotection, Han and Chesney [47,48] found that upregulation of the taurine transporter significantly reduces the rate of LLC-PK1 cell and renal apoptosis initiated by cisplatin. During the process of apoptosis, apoptotic cells, such as those treated by cisplatin, undergo an exaggerated activation of the regulatory volume decrease in which taurine effluxes the cell. If the regulatory volume decrease is disrupted by preloading the cells with taurine several apoptotic steps, such as DNA fragmentation and apoptotic cell shrinkage, are prevented [50]. Lang *et al.*[50] found that while taurine loading did not prevent early apoptotic events, such as caspase activation, it blocked the progression of the apoptotic cascade beyond the cell shrinkage step. Based on these findings, Ito *et al.*[51] proposed that Adriamycin-mediated cardiotoxicity might be caused in part by taurine deficiency. Not only does Adriamycin indirectly downregulate the expression of the taurine transporter [46,51], but taurine therapy minimizes the cardiotoxicity of Adriamycin [51,52].

Although taurine loss during apoptotis has an adverse effect on the cell, the loss of taurine during a normal regulatory volume decrease can benefit the hyperosmotically stressed cell by serving as a safety valve to prevent membrane damage caused by excessive cell swelling. This occurs in the ischemic heart, which accumulates osmolytes, such as lactate, phosphate and sodium. Indeed, Allo *et al.*[53] reported that drug-induced taurine depletion reduces the taurine gradient across the cell membrane of the ischemic heart, a factor that contributes to the observed protection against ischemic injury. Other factors contributing to the observed cytoprotection are alterations in the levels of cationic osmolytes, such as Na^+^, and activation of cytoprotective protein kinase pathways [54,55].

While osmotic stress affects taurine transport, changes in taurine levels can also affect the transport of other ions through alterations in osmotic stress. One such change occurs during pharmacological elevations in extracellular taurine, which like cell shrinkage, enhances Na^+^-Ca^2+^ exchange activity and elevates [Ca^2+^]_i_[56]. Similarly, elevations in intracellular taurine often mimic osmotic-mediated cell swelling. Among the transporters that are stimulated by both cell swelling and elevations in intracellular taurine are the fast Na^+^ current and Cl^-^ current [56]. Suleiman *et al.*[57] attributed the interaction between taurine and Na^+^ movement to the cotransport of taurine and Na^+^ via the taurine transporter. Because hyperosmotic stress promotes the upregulation of the taurine transporter, while a rise in intracellular taurine simultaneously increases the osmotic load and mass action-mediated efflux of taurine and Na^+^ via the taurine transporter, the association between the effects of taurine and certain types of osmotic stress are predictable. In contrast, there is a poor correlation between osmotic-mediated and taurine-mediated changes in K^+^ current [56].

The link between taurine and Cl^-^ current is intriguing, as the most widely distributed Cl^-^ channel, known as the anion organic osmolyte channel, also conducts taurine [58]. Moreover, a member of the FXYD family of small membrane-spanning proteins, known as phospholemman, forms volume-sensitive channels that conduct anions, such as Cl^-^, as well as the zwitterion, taurine [59]. Because phospholemman is regulated by protein kinases involved in regulatory volume changes, it has been implicated in cell swelling of cardiomyocytes subjected to hypoosmotic challenges. Indeed, reductions in phospholemman expression decrease taurine efflux in astrocytes [60]. However, cardiomyocytes obtained from phospholemman knockout mice swell to the same extent as wild-type mice in response to a hypoosmotic challenge [61]. Moreover, mouse cardiomyocytes do not undergo a characteristic regulatory volume decrease, raising the possibility that phospholemman may only play a subtle role in volume regulation. Nonetheless, phospholemman is closely tied to transporters involved in osmotic regulation. Jia *et al.*[62] found that the heart of phospholemman knockout mice exhibit reductions in Na^+^-K^+^ ATPase activity and increases in ejection fraction. In contrast, overexpression of phospholemman increases contractility and elevates [Ca^2+^]_i_, effects attributed to inhibition of the Na^+^/Ca^2+^ exchanger [63,64]. Thus, there are still many unresolved questions regarding the role of regulatory volume processes and the osmoregulatory actions of taurine in the heart.

### Conjugation reactions of taurine in the heart and skeletal muscle

Taurine is involved in several conjugation reactions, with the most common reactions occurring in the liver. Nonetheless, one of the most important conjugation reactions takes place in the mitochondria of extrahepatic cells and has a major impact on mitochondrial function [6,8]. The formation of 5-taurinomethyl uridine in the anticodon wobble position of mitochondrial tRNA^Leu(UUR)^ increases UUG translation although the translation of UUA is unaffected [7]. A direct cause-effect relationship between a deficiency in 5-taurinomethyluridine and mitochondrial diseases has been proposed but not definitively established. A major complication is that patients with symptoms of myopathy, encephalopathy, lactic acidosis and stroke-like episodes contain several mutations, only one resulting in a reduction in 5-taurinomethyluridine content [5]. Based on the potential role of 5-taurinomethyluridine in modulating mitochondrial protein synthesis, Schaffer *et al.*[8] proposed a mechanism for the regulation of reactive oxygen species production by taurine. They predict that diminished rates of 5-taurinomethyluridine arising from taurine deficiency reduce the expression of mitochondrial encoded proteins. Because these proteins are functional subunits of respiratory chain complexes, reduced expression invariably decrease the activity of the complexes. As flux of electrons through the respiratory chain diminishes, there is a risk that electrons will be diverted from the electron transport chain to other acceptors, such as oxygen. The major source of electrons involved in mitochondrial ROS generation comes from complexes 1 and 3 [65]. By elevating the expression of mitochondrial encoded proteins, taurine ensures a smooth coupling between the delivery of reducing equivalents into the electron transport chain, flux of reducing equivalents through the respiratory chain and ATP production by oxidative phosphorylation.

### Can intracellular taurine provide protection against oxidative stress?

The literature is awash with studies claiming that taurine is an “antioxidant.” Yet Arouma *et al.*[66] have clearly shown that taurine is incapable of directly scavenging the classical reactive oxygen species (ROS). The only reactive species that is directly neutralized by taurine is HOCl, which is converted to N-chlorotaurine [4]. Because N-chlorotaurine is less toxic than HOCl, the neutralization of HOCl by taurine might limit myocardial damage caused by neutrophils [4]. This led to the suggestion that the formation of N-chlorotaurine might be the most important antioxidant activity of taurine [67]. However, recent studies have uncovered a key action of taurine could regulate the rate of ROS generation by the mitochondria [8]. This is important because elevated superoxide generation by the mitochondria is capable of initiating the mitochondrial permeability transition, which in turn triggers the apoptotic cascade [68].

Most of the studies that ascribe an antioxidant activity to taurine utilize pharmacological levels of the β-amino acid to minimize oxidative damage. Only a few studies have examined the potentiation of oxidative damage by taurine depletion. In one such study, Harada *et al.*[52] showed that drug-induced taurine deficiency potentiates Adriamycin-induced oxidative stress in the heart, an effect thought to exacerbate the cardiotoxicity of Adriamycin. In a related study, Schaffer *et al.*[69] found that drug-induced taurine deficiency enhances angiotensin II-mediated apoptosis. Although Schaffer *et al.*[69] did not evaluate the effect of taurine deficiency on angiotensin II-mediated oxidative damage, Ricci *et al.*[68] recently found that angiotensin II-mediated apoptosis is largely caused by increased oxidative damage within the mitochondria. These two studies revealing an adverse effect of taurine depletion are seemingly at odds with a study that uncovered a beneficial effect of taurine deficiency against ischemia [53]. However, this apparent contradiction merely emphasizes the multifaceted actions of taurine. While the antioxidant activity of taurine might benefit the hypoxic cardiomyocyte, the dominant effects of taurine depletion are diminished Na^+^ and Ca^2+^ overload and stimulation of the PI 3-kinase/Akt signaling pathway [54,55,70,71].

### Role of taurine in membrane stabilization

In 1973 Huxtable and Bressler [72] found that incubation of isolated sarcoplasmic reticulum with phospholipase C damaged the membrane, leading to reductions in Ca^2+^ transport and ATPase activity that were diminished by addition of taurine to the medium. This observation led the authors to propose that taurine acts as a membrane stabilizer. It was subsequently shown that taurine directly interacts with membranes, presumably by forming an electrostatic interaction between the amino and sulfonic acid groups of taurine and the phosphate and amino or quaternary ammonium groups of the phospholipids, respectively [73]. This interaction appears to cause minor changes in the lipid bilayer, allowing more calcium to bind to the phospholipids [74]. However, based on electron spin resonance, neither polymorphic phase changes nor fluidity changes are induced in the lipid bilayer following acute exposure of isolated membranes with taurine. This led to further characterization of the membrane-linked actions of taurine. One of the most prominent taurine-mediated changes in membrane function was found to be the inhibition of phospholipid N-methyltransferase, an enzyme that catalyzes the conversion of phosphatidylethanolamine (PE) to phosphatidylcholine (PC) [75]. This reaction is important because taurine levels regulate the ethanolamine:choline ratio of some membranes [76]. In the biological membrane, PE is preferentially localized to the outer membrane leaflet where it assumes a bilayer structure while PC is a hexagonal former and is preferentially localized to the inner leaflet of the membrane. Thus, changes in the PE/PC ratio have a dramatic influence on the structure of biological membranes, which in turn alters both membrane fluidity and the activity of membrane enzymes and transporters [73].

### Are taurine-mediated alterations in protein phosphorylation important physiological functions in heart and skeletal muscle?

The idea that taurine is capable of altering the phosphorylation of key proteins was initially demonstrated in hearts and retina of taurine treated and drug-induced taurine depleted rats [77]. In the heart, the taurine-dependent phosphorylation of a 44 kDa protein is promoted by reductions in taurine levels but diminished by taurine treatment. The effect of taurine depletion on the 44 kDa protein, which was subsequently identified as pyruvate dehydrogenase, is consistent with the metabolic changes mediated by drug-mediated taurine depletion of rat heart [78]. In accordance with the known inhibition of pyruvate dehydrogenase by protein phosphorylation, Mozaffari *et al.*[78] found that taurine depletion led to a significant elevation of lactate and pyruvate production despite the reduction in pyruvate utilization by the citric acid cycle. The enzyme involved in the phosphorylation of pyruvate dehydrogenase in the taurine deficient heart was not identified, but the response to inhibitors ruled out any involvement of cAMP and cGMP dependent protein kinases [79].

Protein kinase C is also closely related to the osmoregulatory activity of taurine. According to Liu *et al.*[80] hypoosmotic stress promotes the translocation of selective protein kinase C isoforms (PKCα, PKCε, and PKCζ) to specific membranes, an event associated with their activation. Interestingly, taurine treatment also leads to the activation of the same three protein kinase C isoforms [81]. The activation of these same protein kinase C isoforms contributes to the osmoregulatory actions of taurine by activating a signaling pathway in which an early event is the stimulation of NADPH oxidase. In the hypoosmotically stressed cell, NADPH oxidase-derived ROS inhibits protein tyrosine phosphatase 1B, which potentiates swelling-induced taurine release [82,83].

Tyrosine kinases represent another group of protein kinases that are regulated by osmotic stress. Following a hypoosmotic insult, tyrosine kinases activate a downstream target, PI 3-kinase, whose activation stimulates the phosphorylation of Akt [84]. Upregulation of the PI 3-kinase/Akt pathway is cytoprotective, rendering the cell resistant to pathological insults, such as hypoxia, osmotic imbalances, energy deficiency and cationic overload. Drug-induced taurine deficiency also leads to the activation of the PI 3-kinase/Akt pathway, rendering the cell resistant to hypoxia-mediated cell death [54]. Although the beneficial effect of taurine depletion against hypoxic injury has been attributed to diminished accumulation of Na^+^ and Ca^2+^[55,57], the stimulation of protein kinase-linked cytoprotective pathways, such as the PI 3-kinase/Akt pathway and PKCε associated cytoprotective pathways, are also likely candidates for the cytoprotection. Because the effects of taurine depletion resemble the potent cytoprotective procedure referred to as ischemic preconditioning [53,54,71], the protein kinase-linked pathways warrant further consideration.

Recent studies reveal that the phosphorylation state of two proteins involved in excitation-contraction coupling is affected by taurine depletion (see section on contractile function). However, the protein kinases affecting these phosphoproteins as well as the importance of these phosphorylation changes in the taurine transporter knockout mice remain to be determined.

## Competing interests

The authors declare that they have no competing interests.
